# Isolation, Characterization and Whole-Genome Analysis of *Paenibacillus andongensis* sp.nov. from Korean Soil

**DOI:** 10.4014/jmb.2211.11033

**Published:** 2023-03-08

**Authors:** Yong Guan, Zhun Li, Yoon-Ho Kang, Mi-Kyung Lee

**Affiliations:** 1 Biological Resource Center, Korean Collection for Type Cultures (KCTC), Korea Research Institute of Bioscience and Biotechnology, Jeongeup 56212, Republic of Korea; 2 Department of Integrative Food, Bioscience and Biotechnology, Chonnam National University, Gwangju 61186, Republic of Korea; 3 Department of Environmental Biotechnology, KRIBB School of Biotechnology, University of Science and Technology (UST), Daejeon, 34113, Republic of Korea; 4 Water Environment Research Department, National Institute of Environmental Research, Incheon 22689, Republic of Korea

**Keywords:** Genome analysis, taxonomy, *Paenibacillus*, secondary metabolites, novel species

## Abstract

The genus *Paenibacillus* contains a variety of biologically active compounds that have potential applications in a range of fields, including medicine, agriculture, and livestock, playing an important role in the health and economy of society. Our study focused on the bacterium SS4^T^ (KCTC 43402^T^ = GDMCC 1.3498^T^), which was characterized using a polyphasic taxonomic approach. This strain was analyzed using antiSMASH, BAGEL4, and PRISM to predict the secondary metabolites. Lassopeptide clusters were found using all three analysis methods, with the possibility of secretion. Additionally, PRISM found three biosynthetic gene clusters (BGC) and predicted the structure of the product. Genome analysis indicated that glucoamylase is present in SS4^T^. 16S rRNA sequence analysis showed that strain SS4^T^ most closely resembled *Paenibacillus marchantiophytorum* DSM 29850^T^ (98.22%), *Paenibacillus nebraskensis* JJ-59^T^ (98.19%), and *Paenibacillus aceris* KCTC 13870^T^ (98.08%). Analysis of the 16S rRNA gene sequences and Type Strain Genome Server (TYGS) analysis revealed that SS4^T^ belongs to the genus *Paenibacillus* based on the results of the phylogenetic analysis. As a result of the matrix-assisted laser desorption/ionization-time-of-flight mass spectrometry (MALDI-TOF/MS) results, SS4^T^ was determined to belong to the genus *Paenibacillus*. Comparing *P. marchantiophytorum* DSM 29850^T^ with average nucleotide identity (ANI 78.97%) and digital DNA-DNA hybridization (dDDH 23%) revealed values that were all less than the threshold for bacterial species differentiation. The results of this study suggest that strain SS4^T^ can be classified as a *Paenibacillus andongensis* species and is a novel member of the genus *Paenibacillus*.

## Introduction

The characteristics of the genus *Paenibacillus* was first reported by Ash *et al*. based on an analysis of 16S rRNA sequences of group 3 *Bacilli* [1]; this was later amended by Shida *et al*. [2] and Behrendt *et al*. [3]. Members of this genus are capable of generating stress-resistant spores and possess unique physiological characteristics [4]. They can produce a variety of bioactive substances and they exist in different habitats such as soil and plant roots. In addition to treating diseases caused by bacteria and fungi, they have several applications in the medical field, daily life, and agriculture [5-7]. Many *Paenibacillus* species produce antimicrobial compounds that are useful as medicine or pesticides or enzymes that are useful in bioremediation or chemical production. For example, some *Paenibacillus* species have been shown to promote the growth of plants, such as maize[8] and pumpkin [9]. Some species are also capable of nitrogen-fixing [10]. At present, over 200 species have been isolated, identified, and described as members of the genus *Paenibacillus* (https://lpsn.dsmz.de/search?word=paenibacillus+). The genus *Paenibacillus* comprises gram-positive bacteria that are rod-shaped with oxidase-positive properties. Anteiso-C_15:0_, C_16:0_, and iso-C_16:0_ are the most common cellular fatty acids. Phosphatidylglycerol, diphosphatidylglycerol, and phosphatidylethanolamine are the major polar lipids [11], and MK-7 is the quinone present. Generally, the DNA G+C content ranges from 39–59 mol% [12].

Recently, we isolated a novel bacterium (named as strain SS4^T^) from a soil sample. MALDI-TOF MS analysis confirmed that strain SS4^T^ belongs to the genus *Paenibacillus*. In this study, we characterized this novel strain SS4^T^ based on the results of phenotypic, genotypic, chemotaxonomic, and phylogenetic analyses. In addition, we predicted the secondary metabolites based on the bioinformatics analysis.

## Materials and Methods

### Isolation of the Bacterial Strain and Culture

Soil samples for our analysis were collected from Andong, Korea (36°35'39"N, 128°49'16"E). Using sterile 50 ml tubes, we collected soil samples and stored them in a refrigerator at the laboratory at 4°C. Isolation of the strain was achieved by adding two grams of soil into a sterile 15 ml tube containing a ten-fold dilution of phosphate-buffered saline (PBS) buffer and spreading it on fresh Reasoner's 2A (R2A) agar plates. After three days of incubation at 25°C, several colonies were transferred to fresh R2A plates to purify the cultures by streaking until isolates were obtained. One of these purified isolates represented a novel species and was designated SS4^T^. The SS4^T^ strain was stored at -80°C in 20% glycerol.

### 16S rRNA Gene Sequencing, BLAST and Phylogenetic Analysis

Genomic DNA was extracted using a PowerSoil Pro DNA Isolation Kit (Cat:47014; Qiagen, USA). 16S rRNA gene sequencing was performed using two universal primers: 518F (5-CCA GCA GCC GCG GTA ATA C-3) and 805R (5-GAC TAC CAG GGT ATC TAA TC-3). We compiled the complete 16S rRNA sequence using the BioEdit program [13] and submitted it to GenBank. Clustal W [14] was used to align the sequences of SS4^T^ with that of closely related strains. We generated phylogenetic trees for SS4^T^ and closely related strains using the neighbor-joining [15], maximum-likelihood [16], and minimum-evolution [17] methods using Kimura's two-parameter model [18]. Phylogenetic analyses were conducted using Molecular Evolutionary Genetics Analysis (MEGA) software (version 7.0) [19] with 1000 bootstrap iterations [20]. The Basic Local Alignment Search Tool (BLAST) program was used to search the GenBank database for homology with the 16S rRNA gene sequences obtained in this study [21].

### Genomic Analyses

Genomic DNA was extracted using the PowerSoil^®^ Pro DNA Isolation Kit (Cat:47014; Qiagen). The DNA quality was checked with agarose gel (0.8%), and the integrity and quality were also determined using Qubit (NANODPOP 2000). Sequencing was performed using an Illumina NovaSeq 6000 sequencer. Simultaneously, nanopore sequencing of the genomic DNA was further performed using the MinION platform from Oxford Nanopore Technologies (ONT). Sequencing libraries were prepared using a ligation sequencing kit (SQK-LSK109; ONT) following the manufacturer’s handbook (version RPB_9059_v1_revC_08Mar2018) with SPRI bead clean-up (AMPure XT beads; Beckman Coulter, USA). Sequencing was performed as multiplex runs on a MinION with MinKnow v1.15.1 using FLO-MIN106 R9.4 flow cells. Average nucleotide identity (ANI) values were derived from the ANI tool (www.ezbiocloud.net/tools/ani) [22], and the genome-to-genome distance calculation web server (http://ggdc.dsmz.de/distcalc2.php) [23] was used to determine the DNA–DNA hybridization (DDH) value. The OrthoANI value was calculated using the standalone Orthologous Average Nucleotide Identity (OAT) software (version 0.93.1) [24]. The genomic sequence of SS4^T^ strain was uploaded to the Type Strain Genome Server (TYGS)—a free bioinformatics platform for a whole genome-based taxonomic analysis (https://tygs.dsmz.de) [25]. The phylogenomic tree was reconstructed using FastME 2.1.6.1, including SPR post-processing from the genome BLAST distance phylogeny (GBDP) [26]. Branch support was inferred from 100 pseudo-bootstrap replicates each. The same pipeline was used to annotate all genomes including those from the present study to secure a comparison. Several clusters, including all newly sequenced genomes in the present study, were tested using ROARY to identify all accessory genes unique to each genome. Functional genes within each genome were annotated using Kyoto Encyclopedia of Genes and Genomes (KEGG) and deciphered to pathways using KEGG Decoder [27] and KEGG-Expander (https://github.com/bjtully/BioData/tree/master/KEGGDecoder). Rapid Annotation of microbial genomes using Subsystems Technology (RAST) was also used to validate the annotations, particularly the subsystems [28]. The online software antiSMASH [29] was used to analyze the gene clusters for secondary metabolites. PRISM was used to analyze gene clusters for nonribosomal peptides and polyketide compounds [30]. A potential bacteriocin was identified and analyzed using the online software BAGEL 4 [31].

### Morphology, Biochemical and Physiologic Characteristics

To determine the cell shape, the cells were desiccated with a critical point dryer (SPI-Dry Conventional Critical Point Dryer), coated with gold using a Safematic CCU-010HV high-vacuum sputter, and examined with a scanning electron microscope (SEM). A Gram staining kit (Bio Mérieux, France) was used to determine Gram staining under a light microscope. Cell motility was determined by observing the growth of strain SS4^T^ in a semi-solid R2A medium containing 0.5% agar after incubation at 25°C for 5 days. The growth on R2A was examined at different temperatures (4, 15, 20, 25, 30, 35, and 40°C) for four days. The strain was cultured at different pH (4.0–10.0, at increments of 1.0 pH unit) to determine the pH tolerances and optimal pH for growth. Cell optical density (OD) values were monitored at different salt concentrations (0.5–5%) to estimate salt tolerance. Anaerobic test was conducted under anaerobic conditions: 7% CO_2_, 86% N_2_, and 7% H_2_. The catalase test was conducted using a catalase reagent (BioMérieux). The oxidase test was confirmed based on the production of a blue color using an oxidase reagent (BioMérieux). Enzyme activities and biochemical properties were determined using API ZYM and ZPY 20NE. To determine the strain amounts of IAA produced using the Salkowski's reagent (2% 0.5 FeCl_3_ in 35% HClO_4_ solution) and kept in the dark condition. The OD value was detected at 530 nm after 30 min. For analysis of cellular fatty acid, the cells were saponified, methylated, and extracted using the Microbial Identification System (MIDI; Microbial ID Inc., Newark, DE, USA) [32] using instructions provided by the manufacturer [33], and the extracts were identified using gas chromatography (GC-210; Shimadzu, Japan) and SherlockTM Chromatographic Analysis System software package with the aerobic database version 6.1. The biomass was freeze-dried to analyzed the compounds for polar lipids and identify them. We used TLC silica gel 60 F254 (20x20) and dyes that included 50% H_2_SO_4_, molybdenum (Sigma-Aldrich, USA), and 0.1% ninhydrin (Sigma-Aldrich) to identify total lipids, phospholipids, and amino lipids, respectively. A flow rate of 1 ml/min was utilized in reverse-phase high performance liquid chromatography (HPLC) to detect quinone extracted from the biomass of the SS4^T^ strain and other closely related strains.

### Maldi-Tof MS

Strain SS4^T^ and the closely related strains were cultured on R2A, Tryptic Soy Agar (TSA), and nutrient plates at 25°C for 2 days. Each colony was analyzed thrice by following the instructions in the HCCA/formic acid (70%) extraction manual provided by Bruker Daltonics for MALDI-TOF MS analysis of a single colony. In this study, spectral measurements were performed using a Flexcontrol (version 3.4) instrument with a mass range of 2,000–20,000 m/z, and the data were analyzed using FlexAnalysis and MALDI Biotyper Compass Explorer (version 4.1.100).

## Results and Discussion

### 16S rRNA Gene Sequence Analysis

**Fig. 1:**
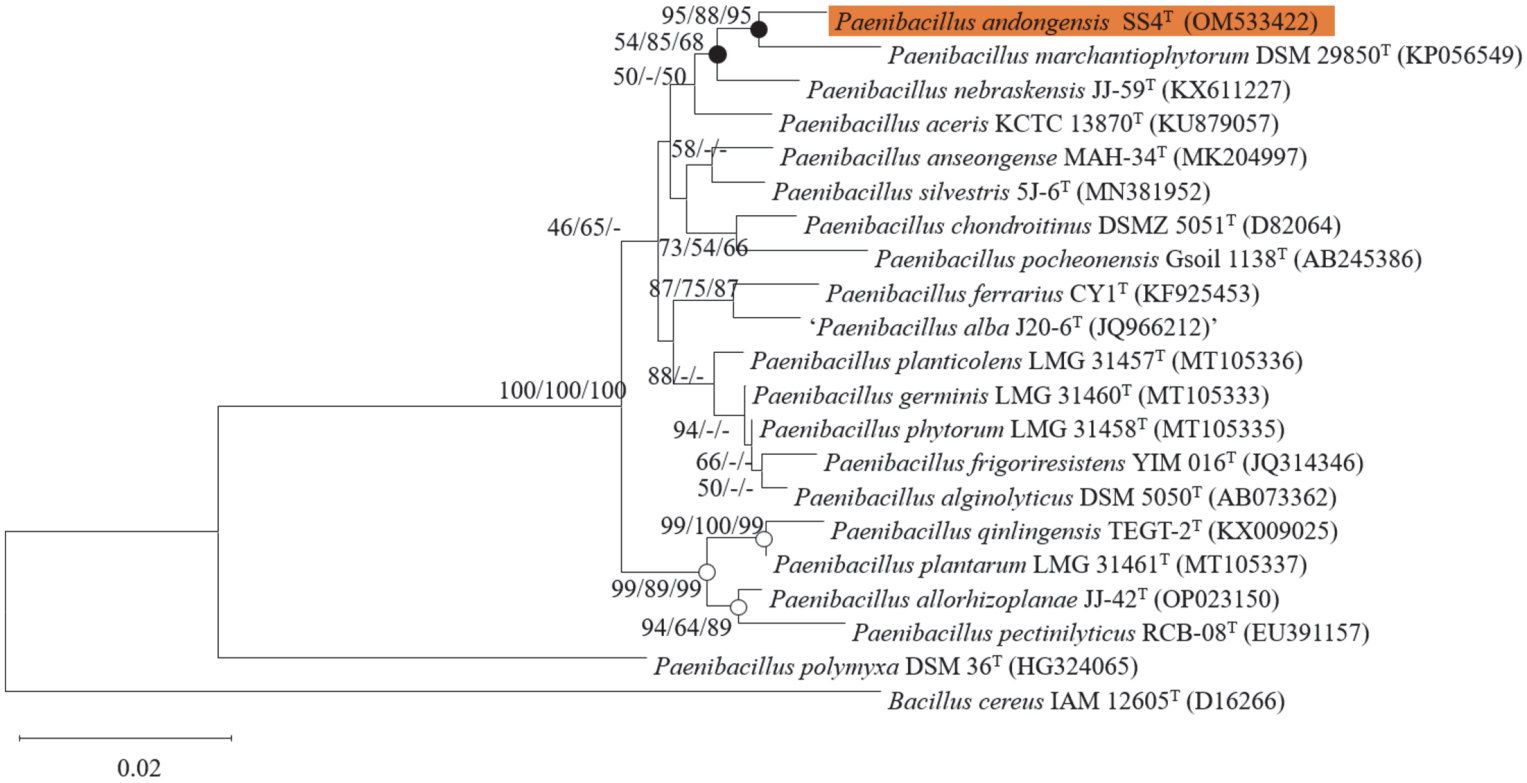
Minimum-evolution tree based on the 16S rRNA gene sequences. Bootstrap support values (1000 replications) over 50% are shown at nodes. Bootstrap values from minimum-evolution, neighbor-joining and maximum-likelihood analyses are shown (NJ/ML/ME). Closed circles indicate that the corresponding nodes were also recovered in trees generated with the ML and ME methods. Open circles indicate that the corresponding nodes were recovered in the tree generated with the ME, ML and NJ methods. *Bacillus cereus* was used as an outgroup in this tree. Scale bar=0.02 nucleotide substitutions per site.

Comparative analysis of 16S rRNA (1,490 bp) gene sequences showed that SS4^T^ strain has the highest similarity to *P. marchantiophytorm* DSM 29850^T^ (98.22%), *P. nebraskensis* JJ-59 (98.19%), *P. aceris* KCTC 13870^T^ (98.08%), *P. frigoriresistens* DSM 25554^T^ (97.61%), *P. chondroitinus* DSM 5051^T^ (97.55%), and *P. pocheonensis* KCTC 13941^T^ (97.39%) in EzBioCloud database. In addition, comparative 16S rRNA sequence analysis using BLAST showed that SS4^T^ strain showed 98.18%, 97.80%, and 97.45% 16S rRNA sequence homology to *P. marchantiophytorum* (Accession no. NR_148618.1), *P. nebraskensis* (Accession no. NR_159223.1), and *P. aceris* (Accession no. NR_156841.1), respectively (Table S1). These values were less than the value of 98.65% required for declaring a novel species [34]. The phylogenetic tree revealed that the SS4^T^ strain is closely related to *P. marchantiophytorum* DSM 29850^T^ (Fig. 1). Based on the 16S rRNA gene sequence analysis, SS4^T^ strain can be declared as a novel species of the genus *Paenibacillus*.

### Genomic Analyses

**Fig. 2:**
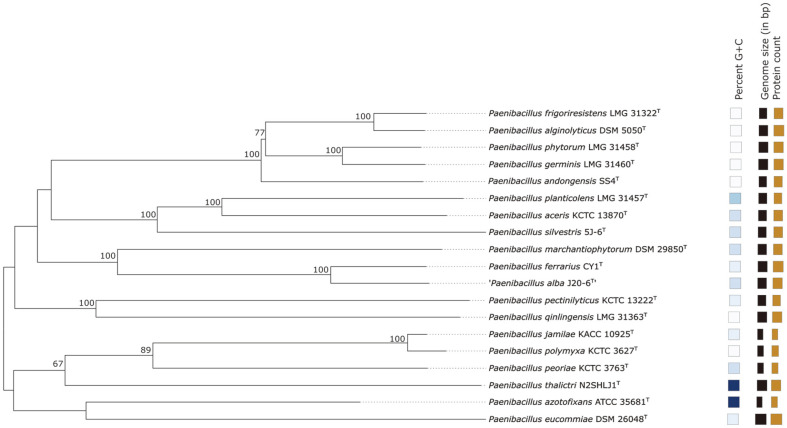
Tree inferred with FastME 2.1.6.1 from GBDP distances calculated from genome sequences. The branch lengths are scaled in terms of GBDP distance formula d5. The numbers above branches are GBDP pseudo-bootstrap support values > 60% from 100 replications, with an average branch support of 74.2%. The tree was rooted at the midpoint. Leaf labels with different colors indicate percent GC (blue), genome size (Black) and protein count (Brown).

The phylogenomic tree constructed based on TYGS analysis revealed the relationship between SS4^T^ strain and the closely related type strains (Fig. 2). It also showed that SS4^T^ strain was placed in a species branch different from that of the other *Paenibacillus* species. Comparison of the genomic dDDH values of SS4^T^ strain and its closest related strain yielded a value of 23%, which was within the cut-off value determined as a threshold for novel species [23]. The ANI value between SS4^T^ strain and its closest relatives reached 78.97% (Table S2), and this value was less than the 95–96% threshold for novel species description. OrthoANI values between SS4^T^ strain and *P. marchantiophytorm* DSM 29850^T^ reached 78.49% [35-38].

**Fig. 3:**
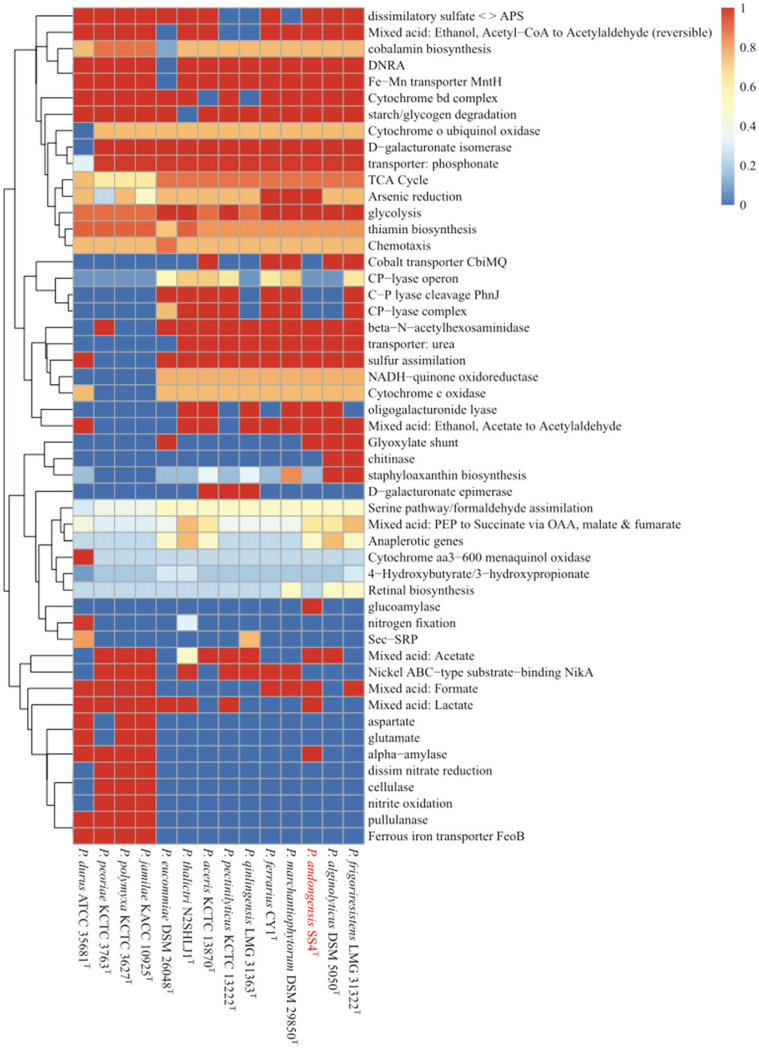
The heatmap of discriminated metabolic pathways within the genus *Paenibacillus* with 13 representative genomes including *Paenibacillus andongensis* SS4^T^. The cell indicates the completeness of each pathway referring to annotations using KEGG.

The genome of SS4^T^ strain contained 6,909 genes, with a total length of 7,639,302 bp. There were 107 tRNAs, 37 rRNAs (5S, 16S, and 23S), and 1 tmRNA. Based on the whole-genome sequence, the DNA G+C content was found to be 44.96%. In addition, there were 6,669 CDS. We also note that only SS4^T^ can produce glucoamylase and alpha-amylase compared to the closest strain. In addition, glycolytic, mixed acid and galactose-oligosaccharide lyases can be produced (Fig. 3). Based on our genomic findings, we further confirmed that SS4^T^ was capable of producing glucoamylase (Fig. S1). Glucoamylase is a high-demand commercial biocatalyst in the food industry, and its demand far exceeds that of other enzymes [39]. Based on the analysis of the genome sequence with antiSMASH version 6, there are five gene clusters were predicted. One of the five gene clusters showed 100%similarity with known biosynthetic gene clusters (BGC), another showed 31% similarity, and the last cluster showed less than 50% similarity, which may indicate that SS4^T^ strain is capable of producing new natural products. Five clusters were found between NRPS, proteusin, lasso peptide, and two others BGCs (Fig. S5A). As cluster 1 is a hybrid cluster of NRPS/PKS, the NRPS/PKS product and its polymer properties were predicted for the polymer cluster (Figs. S5B and S5C). The metabolites detected among SS4^T^ and its closely related strains were proteusin, lasso peptide, thioamide-NRP, terpene, RiPP-like, NRPS, LAP/RiPP-like, and siderophore (Table S4). More proteusin and lasso peptide metabolites have been found in *Paenibacillus* strains. Additionally, siderophore-type metabolites were detected only in the type strain *P. aceris* KCTC 13870^T^. Additionally, thioamide-NRP-type metabolites were detected only in the type strain *P. marchantiophytorm* DSM 29850^T^ (Table S4). A biosynthetic cluster containing RRE was detected in the SS4^T^ strain.

During the same time period, we compared the types of secondary metabolites produced by similar bacteria, among which lasso peptide was the most common type and reached 100% (Table S4). In the NRPS/PKS products, we found 1059 peptides based on the Norine database, whereas only 620 peptides were found in the *P. marchantiophytorm* DSM 29850^T^. Several peptides were identified, including antimicrobials, protease inhibitors, surfactants, siderophores, and toxins (Table S5).

We used BAGEL4 to visualize prokaryotic genomes for ribosomal synthesis, post-translationally modified polypeptides (RiPPs), and bacteriocin-producing gene clusters. An analysis of BAGEL4 found two AOIs (area of interest), one starting at 6,550,994 and ending at 6,570,994, classified as lasso peptides (Fig. S6A), and the other starting at 6,119,750 and ending at 6,139,750, classified as LAPs (Fig. S6B). PRISM is an algorithm used to predict natural product structures based on microbial genomes. Using a microbial genome sequence, we identified biosynthetic gene clusters and generated combinatorial libraries of the predicted structures. PRISM analysis revealed three BGC, and the predicted polypeptides were nonribosomal peptides, polyketides, and lasso peptides. Based on the process of biosynthetic assembly (Figs. S7A and S7B) and predicted product structure, clusters 1 and 3 had six and three structures, respectively, whereas cluster 2 did not have any.

KEGG analysis revealed the glucoamylase metabolic pathway, and we confirmed that strain SS4^T^ could produce glucoamylase. Glucoamylase is one of the most popular biocatalysts in the food industry and is more popular than other enzymes [40]. This study demonstrates that this novel species has the potential to produce antimicrobial compounds and glucoamylase.

### Morphological and Biochemical Features

This strain is aerobic, non-motile, positive for oxidase and catalase, and gram-positive. Its rod-shaped cells lack flagella and have a cell size in the range of 2.18–2.35 μm × 0.27–0.29 μm (Fig. S2). The ideal tolerance range for pH is 6.0–8.0, temperature is 15–30°C, and salt is 0.5–2% (w/v; optimum, 0.5%). Using the API 20NE kit, SS4^T^ strain could be used with multiple substrates and could be distinguished from the closest related strains and enzyme activities of API ZYM (Table S3). The SS4^T^ strain was found to utilize tryptophan and produce indole acetic acid (IAA) at a concentration of 90 μg/ml. SS4^T^ strain was mainly composed of anteiso-C_15:0_ and iso-C_16:0_, a fatty acid profile that is typical to the genus *Paenibacillus* (Table 1). For example, *P. marchantiophytorm* DSM 29850^T^ is composed mainly of anteiso-C_15:0_ (71.71%). Additionally, SS4^T^ strain was distinguished from *P. marchantiophytorm* DSM 29850^T^ by higher levels of anteiso-C_15:0_, anteiso-C_17:0_ and an extra C_16:1_ w7c alcohol (Table 1). The major polar lipids were diphosphatidylglycerol (DPG), phosphatidylethanolamine (PE), phosphatidylglycerol (PG), and aminophospholipid (APL) (Fig. S3).

### MALDI-TOF MS Analysis

MALDI-TOF was used to confirm that SS4^T^ strain was of a novel lineage when compared with its nearest-type strains. Based on the result of the cluster analysis of the MALDI-TOF mass spectra, P. andongensis SS4 T and *P. marchantiophytorm* DSM 29850^T^ formed a homogenous cluster separated from *P. aceris* KCTC 13870^T^ and *P. polymyxa* KCTC 3627^T^(Fig. S4).

### Description of *Paenibacillus andongensis* sp. nov.

*Paenibacillus andongensis* (an.dong.en´sis. N.L. masc./fem. adj. *andongensis*, referring to Andong, Korea, from where the type strain was isolated)

Cells are gram-positive rods with rounded ends, aerobic, non-motile, oxidase-positive, catalase-positive, and with cell size in the range 2.18–2.35 μm × 0.27–0.29 μm. Growth occurs in pH range 6.0–8.0 and temperature range 15–30°C with optimum growth at 25°C and pH 7.0. Cells grow well in the presence of 0.5–2% NaCl, and 3%NaCl inhibited the cell-growth. The cells are positive for β-galactosidase activity and negative for alkaline phosphatase, esterase, esterase lipase (C8), lipase (C14), valine arylamidase, cystine arylamidase, trypsin, α-chymotrypsin, β-glucuronidase, N-acetyl-β-glucosaminidase, α-mannosidase, and α-fucosidase activity; positive for indole production and negative for nitrate reduction to nitrite; and positive for utilization of arginine, urea, esculin, gelatin, p-nitrophenyl-β-D-galactopyranoside, glucose, arabinose, mannose, mannitol, N-acetyl-glucosamine, maltose, and gluconate and negative for utilization of caprate, adipate, malate, citrate, and phenyl acetate. The major quinone is MK-7. The polar lipid profiles of SS4^T^ strain comprised diphosphatidylglycerol (DPG), phosphatidylethanolamine (PE), phosphatidylglycerol (PG), aminophospholipid (APL), phospholipid (PL), and one unknown polar lipids. The major fatty acid profiles are anteiso-C_15:0_ (75.59%) and iso-C_16:0_(9.14%). ANI and dDDH values between SS4^T^ and its closest related strain are 78.97% and 23%, respectively. The genomic DNA G + C content is 44.96 mol%.

## Supplemental Materials

Supplementary data for this paper are available on-line only at http://jmb.or.kr.

## Figures and Tables

**Table 1:** Cellular fatty acid composition of strain SS4^T^ and its closely related strains.

Fatty acid	**1**	**2**	**3**	**4**
Saturated				
C_10:0_	-	-	-	0.09
C_12:0_	-	-	-	0.37
C_14:0_	0.54	1.04	2.27	1.43
C_16:0_	1.96	3.63	**14.60**	2.81
C_17:0_	-	-	0.61	-
C_18:0_	-	-	0.79	-
Branched				
Iso-C_14:0_	2.48	2.65	1.26	3.46
Iso-C_13:0_	-	-	-	0.17
Iso-C_15:0_	4.47	6.08	5.40	9.23
Iso-C_16:0_	9.14	9.37	7.05	**10.62**
Iso-C_17:0_	0.65	0.99	2.26	1.32
Anteiso-C_13:0_	-	-	-	0.09
Anteiso-C_15:0_	**75.59**	**71.71**	**54.45**	**66.65**
Anteiso-C_17:0_	4.83	4.53	6.68	3.49
Unsaturated				
C_16:1_ w7c alcohol	0.33	-	-	-
C_16:1_ w11c	-	-	1.51	-
C_18:1_ w9c	-	-	2.41	-
Summed Feature 3	-	-	0.69	**0.27**

Strains: 1, SS4^T^; 2, *P. marchantiophytorum* DSM 29850^T^; 3, *P. polymyxa* KCTC 3627^T^; 4, *P. aceris* KCTC 13870^T^. Summed Feature 3: C_16:1_ w7c and/or C_16:1_ w6c.

## References

[1] Ash C FJ, Wallbanks S, Collins MD (1991). Phylogenetic heterogeneity of the genus *Bacillus* revealed by comparative analysis of smallsubunit-ribosomal RNA sequences. Lett. Appl. Microbiol..

[2] Shida O, Takagi H, Kadowaki K, Nakamura LK, Komagata K (1997). Transfer of *Bacillus alginolyticus*, *Bacillus chondroitinus*, *Bacillus curdlanolyticus*, *Bacillus glucanolyticus*, *Bacillus kobensis*, and *Bacillus thiaminolyticus* to the genus *Paenibacillus* and emended description of the genus *Paenibacillus*. Int. J. Syst. Bacteriol..

[3] Behrendt U, Schumann P, Stieglmeier M, Pukall R, Augustin J, Sproer C (2010). Characterization of heterotrophic nitrifying bacteria with respiratory ammonification and denitrification activity--description of *Paenibacillus uliginis* sp. nov., an inhabitant of fen peat soil and *Paenibacillus purispatii* sp. nov., isolated from a spacecraft assembly clean room. Syst. Appl. Microbiol..

[4] Lal S, Tabacchioni S (2009). Ecology and biotechnological potential of *Paenibacillus polymyxa*: a minireview. Indian J. Microbiol..

[5] Shishido M, Massicotte H. B., Chanway C. P (1996). Effect of plant growth promoting *Bacillus* strains on pine and spruce seedling growth and mycorrhizal infection. Ann. Bot..

[6] Guemouri-Athmani S, Berge O, Bourrain M, Mavingui P, Thiéry JM, Bhatnagar T (2000). Diversity of *Paenibacillus polymyxa* populations in the rhizosphereof wheat (*Triticum durum*) in Algerian soils. Eur. J. Soil Biol..

[7] Niu B, Vater J, Rueckert C, Blom J, Lehmann M, Ru J-J (2013). Polymyxin P is the active principle in suppressing phytopathogenic *Erwinia* spp. by the biocontrol rhizobacterium *Paenibacillus polymyxa* M-1. BMC Microbiol..

[8] Sheela T (2013). Influence of Plant Growth Promoting Rhizobacteria (PGPR) on thegrowth of maize (*Zea mays* L.). Gol. Res. Thoughts 3..

[9] Fürnkranz M, Adam E, Müller H, Grube M, Huss H, Winkler J (2012). Promotion of growth, health and stress tolerance of Styrian oil pumpkins by bacterial endophytes. Eur. J. Plant Pathol..

[10] Weselowski B, Nathoo N, Eastman AW, MacDonald J, Yuan ZC (2016). Isolation, identification and characterization of *Paenibacillus polymyxa* CR1 with potentials for biopesticide, biofertilization, biomass degradation and biofuel production. BMC Microbiol..

[11] Zhou Y, Gao S, Wei DQ, Yang LL, Huang X, He J (2012). *Paenibacillus thermophilus* sp. nov., a novel bacterium isolated from a sediment of hot spring in Fujian province, China. Antonie Van Leeuwenhoek.

[12] Yao R, Wang R, Wang D, Su J, Zheng S, Wang G (2014). *Paenibacillus selenitireducens* sp. nov., a selenite-reducing bacterium isolated from a selenium mineral soil. Int. J. Syst. Evol. Microbiol..

[13] Hall TA (1999). Presented at the Nucleic acids symposium series.

[14] Thompson JD, Higgins DG, Gibson TJ (1994). CLUSTAL W: improving the sensitivity of progressive multiple sequence alignment through sequence weighting, position-specific gap penalties and weight matrix choice. Nucleic Acids Res..

[15] Saitou N, Nei M (1987). The neighbor-joining method: a new method for reconstructing phylogenetic trees. Mol. Biol. Evol..

[16] Felsenstein J (1981). Evolutionary trees from DNA sequences: a maximum likelihood approach. J. Mol. Evol..

[17] Rzhetsky A, Nei M (1992). A simple method for estimating and testing minimum-evolution trees. Mol. Biol. Evol..

[18] Kimura M (1980). A simple method for estimating evolutionary rates of base substitutions through comparative studies of nucleotide sequences. J. Mol. Evol..

[19] Kumar S, Stecher G, Tamura K (2016). MEGA7: molecular evolutionary genetics analysis version 7.0 for bigger datasets. Mol. Biol. Evol..

[20] Felsenstein J (1985). Confidence limits on phylogenies: an approach using the bootstrap. Evolution.

[21] Altschul SF, Gish W, Miller W, Myers EW, Lipman DJ (1990). Basic local alignment search tool. J. Mol. Biol..

[22] Yoon SH, Ha SM, Lim J, Kwon S, Chun J (2017). A large-scale evaluation of algorithms to calculate average nucleotide identity. Antonie Van Leeuwenhoek.

[23] Meier-Kolthoff JP, Auch AF, Klenk HP, Goker M (2013). Genome sequence-based species delimitation with confidence intervals and improved distance functions. BMC Bioinformatics.

[24] Lee I, Ouk Kim Y, Park SC, Chun J (2016). OrthoANI: an improved algorithm and software for calculating average nucleotide identity. Int. J. Syst. Evol. Microbiol..

[25] Meier-Kolthoff JP, Göker M (2019). TYGS is an automated high-throughput platform for state-of-the-art genome-based taxonomy. Nat. Commun..

[26] Lefort V, Desper R, Gascuel O (2015). FastME 2.0: a comprehensive, accurate, and fast distance-based phylogeny inference program. Mol. Biol. Evol..

[27] Graham ED, Heidelberg JF, Tully BJ (2018). Potential for primary productivity in a globally-distributed bacterial phototroph. ISME J..

[28] Overbeek R, Olson R, Pusch GD, Olsen GJ, Davis JJ, Disz T (2014). The SEED and the rapid annotation of microbial genomes using subsystems technology (RAST). Nucleic Acids Res..

[29] Blin K, Shaw S, Kloosterman AM, Charlop-Powers Z, van Wezel GP, Medema MH (2021). antiSMASH 6.0: improving cluster detection and comparison capabilities. Nucleic Acids Res..

[30] Skinnider MA, Merwin NJ, Johnston CW, Magarvey NA (2017). PRISM 3: expanded prediction of natural product chemical structures from microbial genomes. Nucleic Acids Res..

[31] van Heel AJ, de Jong A, Song C, Viel JH, Kok J, Kuipers OP (2018). BAGEL4: a user-friendly web server to thoroughly mine RiPPs and bacteriocins. Nucleic Acids Res..

[32] Piñeiro‐Vidal M, Pazos F, Santos Y (2008). Fatty acid analysis as a chemotaxonomic tool for taxonomic and epidemiological characterization of four fish pathogenic *Tenacibaculum* species. Lett. Appl. Microbiol..

[33] Sasser, Myron (1990). Identification of bacteria by gas chromatogtaphy of cellular fatty acids. MIDI technical note 101.

[34] Kim M, Oh H-S, Park S-C, Chun J (2014). Towards a taxonomic coherence between average nucleotide identity and 16S rRNA gene sequence similarity for species demarcation of prokaryotes. Int. J. Syst. Evol. Microbiol..

[35] Ciufo S, Kannan S, Sharma S, Badretdin A, Clark K, Turner S (2018). Using average nucleotide identity to improve taxonomic assignments in prokaryotic genomes at the NCBI. Int. J. Syst. Evol. Microbiol..

[36] Jain C, Rodriguez RL, Phillippy AM, Konstantinidis KT, Aluru S (2018). High throughput ANI analysis of 90K prokaryotic genomes reveals clear species boundaries. Nat. Commun..

[37] Konstantinidis KT, Tiedje JM (2007). Prokaryotic taxonomy and phylogeny in the genomic era: advancements and challenges ahead. Curr. Opin. Microbiol..

[38] Luo C, Rodriguez RL, Konstantinidis KT (2014). MyTaxa: an advanced taxonomic classifier for genomic and metagenomic sequences. Nucleic Acids Res..

[39] Reilly PJ (1999). Protein engineering of glucoamylase to improve industrial performance - a review. Starch‐Stärke..

[40] Ford C (1999). Improving operating performance of glucoamylase by mutagenesis. Curr. Opin. Biotechnol..

