# Supplementary material for: Isolation, Characterization and Whole-Genome Analysis of Paenibacillus andongensis sp.nov. from Korean Soil
Source: J Microbiol Biotechnol. 2023 Mar 8;33(6):753–9. doi: 10.4014/jmb.2211.11033 (PMC10331935; doi:10.4014/jmb.2211.11033)
Supplement: Supplementary file 1 [file jmb-33-6-753-supple.pdf]

J. Microbiol. Biotechnol.
https://doi.org/10.4014/jmb.2211.11033

Supplementary Figures and Tables

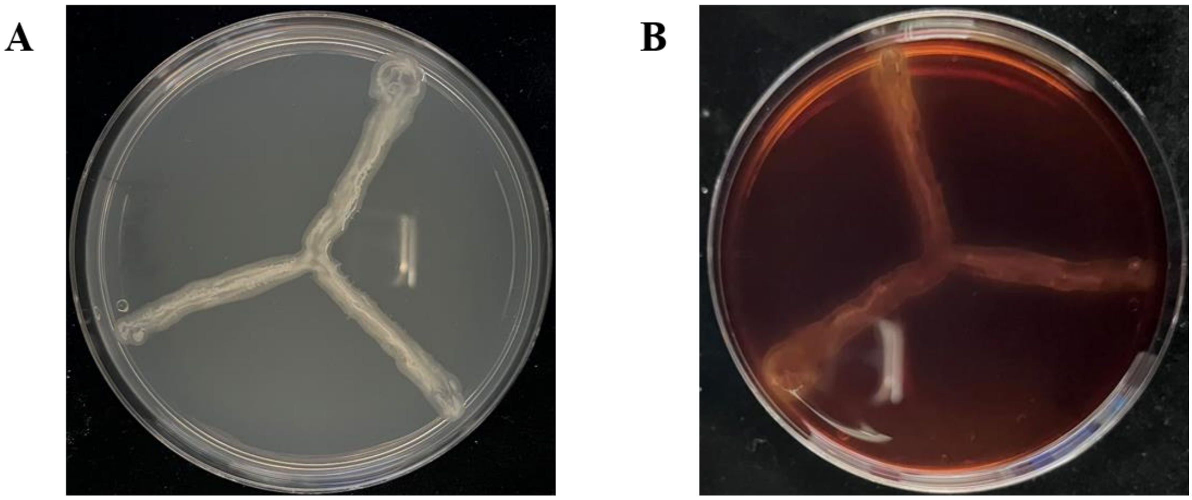

Fig S1. Starch hydrolysis test of SS4^{T} exhibiting zone of clearance.

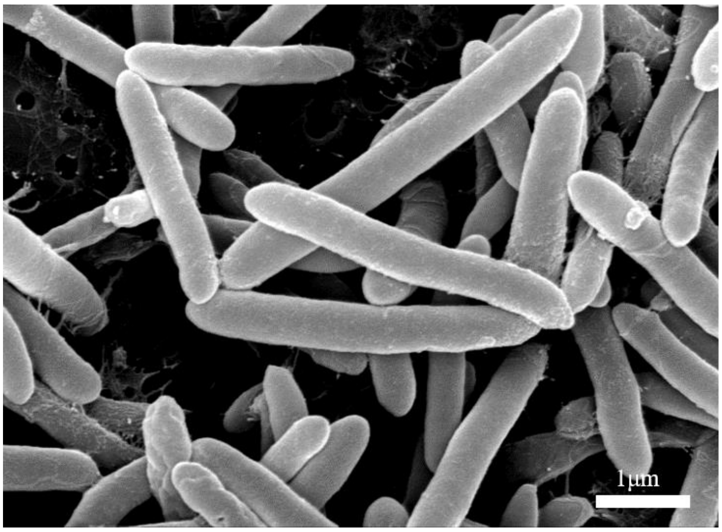

Fig S2. Morphology of strain SS4^{T}, Scale bar, 1μm.

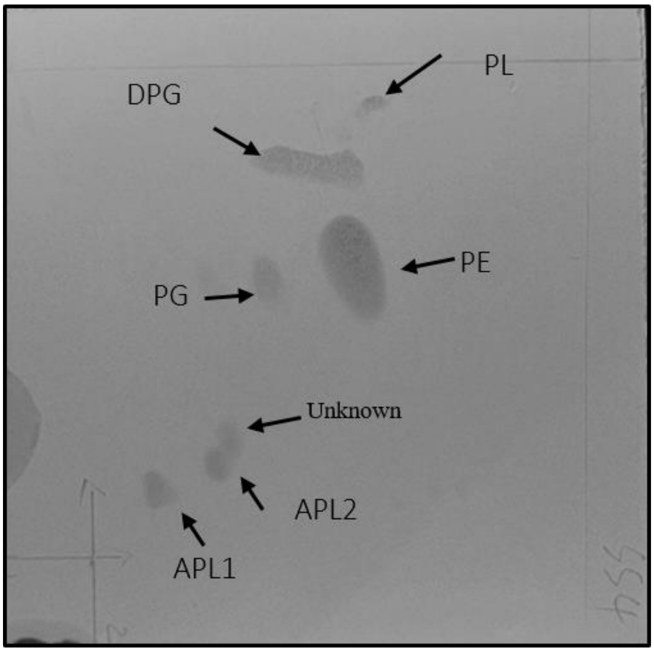

Fig S3. **Two-dimensional thin-layer chromatogram of polar lipid extracts from SS4^T.** DPG, Diphosphatidylglycerol; PE, Phosphatidylethanolamine; PG, Phosphatidylglycerol; APL, Amino phospholipid; PL, Phospholipid.

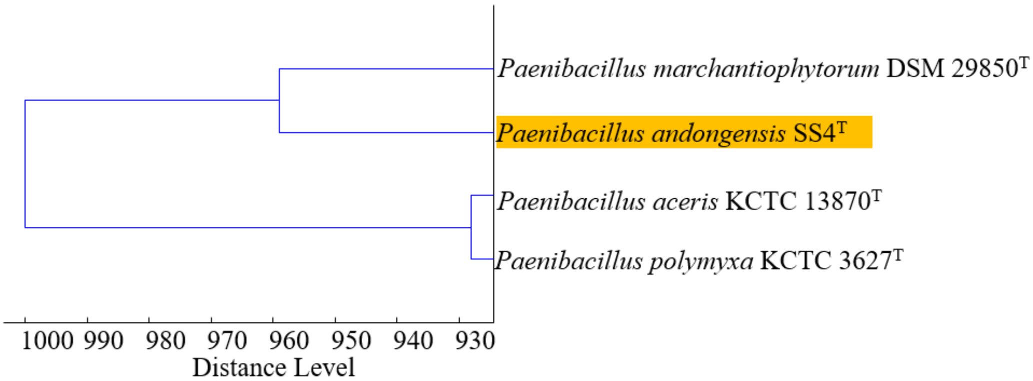

Fig S4. **Cluster analysis of MALDI-TOF MS data of *Paenibacillus andongsoli* SS4^T compared with the close strain of the genus *Paenibacillus.***

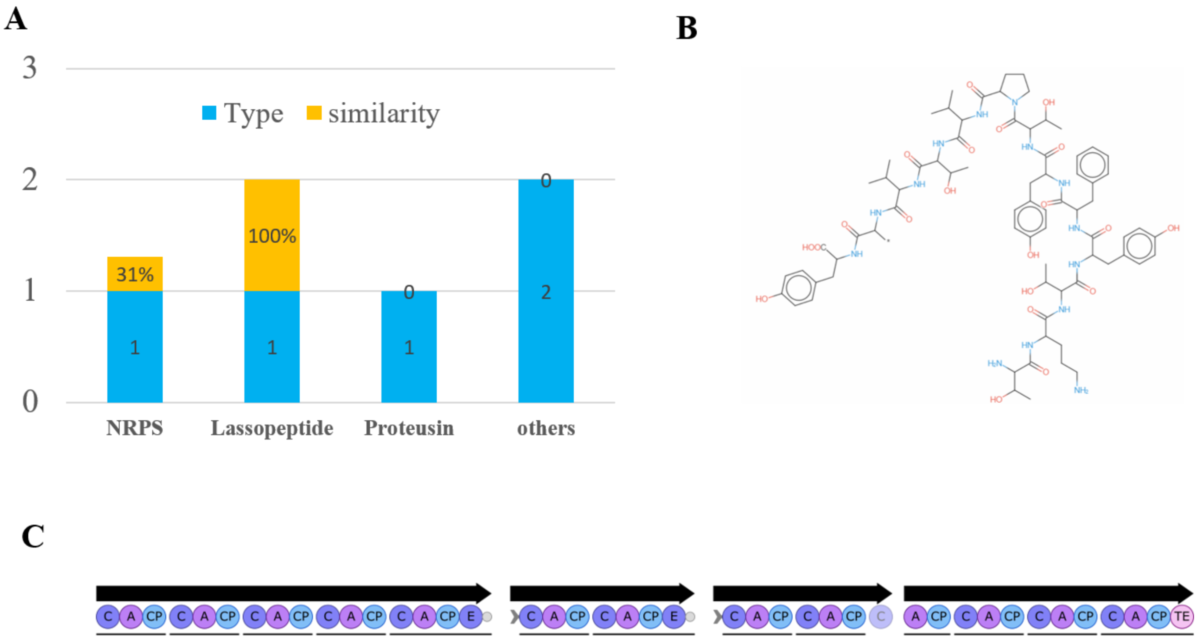

Fig S5. Secondary metabolite analysis of SS4^T. A, Distribution of different types of gene clusters in SS4^T genome; B, Rough prediction of core scaffold based on assumed PKS/NRPS collinearity; C, polymer cluster

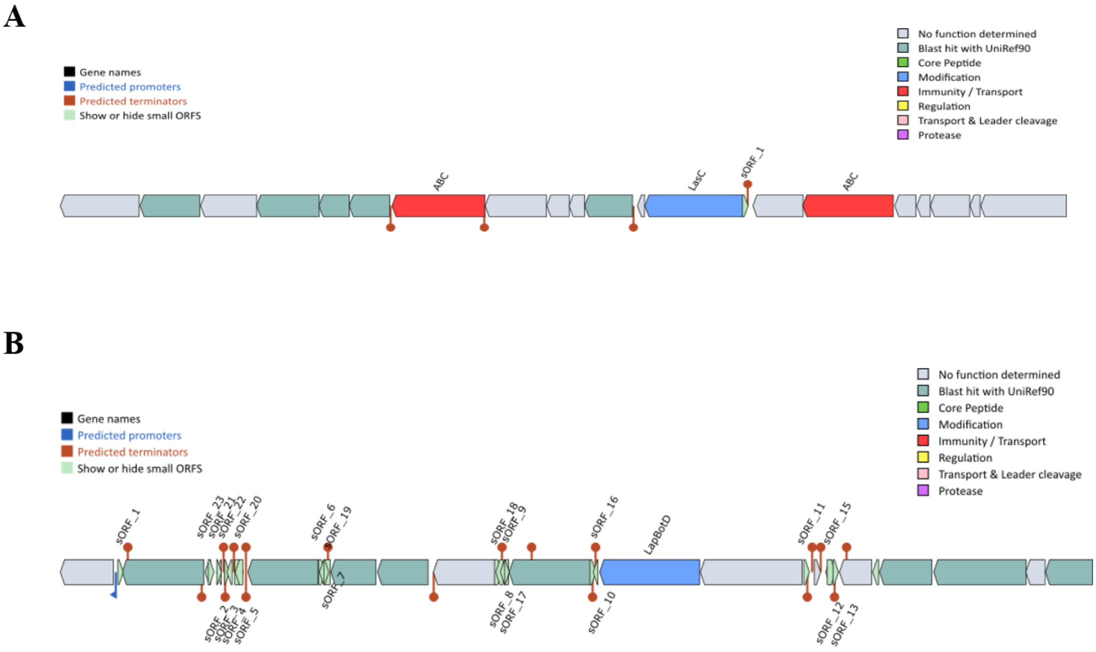

Fig S6. Antimicrobial BGC in the genome sequence of SS4^T. A, Cluster 1, Lassopeptide predicted using BAGEL4; B, Cluster 2, LAPs predicted using BAGEL 4.

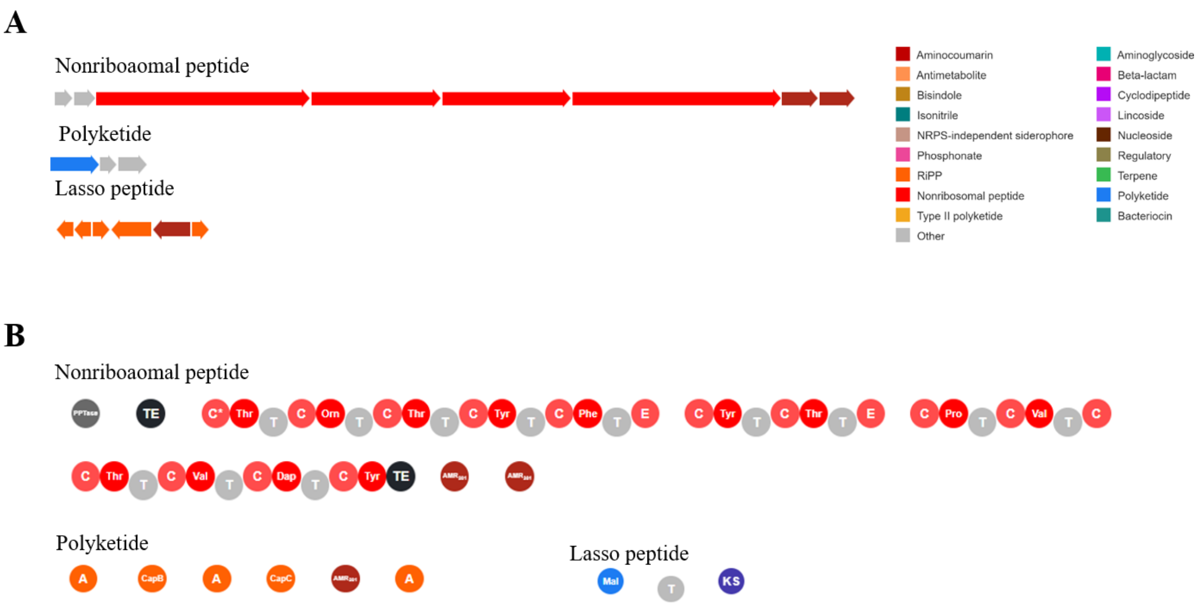

Fig S7. BGC in the genome sequence of SS4^{T}. A, 3 kinds of clusters of natural product were found using PRISM; B, Biosynthetic assembly.

Tables

Table S1. Genus-level identification based on 16S rRNA sequence analysis of the strain SS4^{T} using BLASTA.

16S rRNA BLAST hit (Genus/species/strain)	GenBank accession number	Percent identity
Paenibacillus marchantiophytorum	NR_148618.1	98.19%
Paenibacillus nebraskensis	NR_159223.1	97.80%
Paenibacillus aceris	NR_156841.1	97.45%
Paenibacillus alginolyticus	NR_040893.1	97.51%
Paenibacillus frigoriresistens	NR_109546.1	97.58%
Paenibacillus pocheonensis	NR_112565.1	97.17%
Paenibacillus pectinilyticus	NR_044487.1	97.23%
Paenibacillus chondroitinus	NR_113751.1	96.87%
Paenibacillus qinlingensis	NR_156153.1	96.59%

Table S2. Genomic comparisons between strain SS4^T and its closely phylogenetic neighbors.

GenBank accession number	Strain genome	ANI (%)	dDDH (%)	OrthoANI (%)
GCA_014640555.1	*P. marchantiophytorum* DSM 29850^T	78.97	23	78.49
GCA_017874035.1	*P. aceris* KCTC 13870^T	79.65	23.7	79.14
GCA_004000725.1	*P. alginolyticus* DSM 5050^T	91.38	45.7	91.20
GCA_013266765.1	*P. frigoriresistens* YIM 016^T	91.44	45.9	91.46
GCA_001700435.1	*P. pectinilyticus* RCB-08^T	77.28	21.7	76.73
GCA_015710975.1	*P. polymyxa* KCTC 3627^T	68.59	31.9	67.34

Table S3. Comparison of the biochemical characteristics of SS4^T and closely strains.

**Characteriastics**	**1**	**2**	**3**	**4**
API ZYM				
Acid phosphatase	w	-	w	+
Naphthol-AS-BI-phosphohydrolase	w	w	+	+
a-galactosidase	w	w	+	-
ß-galactosidase	+	w	+	-
a-glucosidase	+	-	+	+
ß-glucosidase	+	-	+	-
N-acetyl-ß-glucosaminidase	-	-	-	+
API 20NE				
NITrate reduction to nitrites	-	-	+	+
tryptophan	+	+	+	-
arginine	+	-	-	-
urea	+	-	-	-
p-nitrophenyl-β-Dgalactopyranoside	+	+	+	-
glucose	+	-	+	-
arabinose	+	-	+	-
mannose	+	-	+	-
mannitol	+	-	+	-
N-acetyl-glucosamine	+	-	-	-
maltose	+	-	-	-
gluconate	+	+	+	-

Strains: 1, SS4^T; 2, *P. marchantiophytorum* DSM 29850^T; 3, *P. polymyxa* KCTC 3627^T; 4, *P. aceris* KCTC 13870^T.+, positive; -, negative; w, weakly positive.

Table S4. Strain SS4^T and its corresponding closest know type strains uing antiSMASH

**Organism**	**Contig/scaffold**	**Type**	**Most similar known cluster**	**Similarity (%)**
P.marchantiophytorum	BMHE01000002.1	proteusin	-	
	BMHE01000005.1	lassopeptide	paeninodin	100
	BMHE01000006.1	Thioamide-NRP	-	
	BMHE01000011.1	terpene	carotenoid	33
	BMHE01000011.2	terpene	-	
	BMHE01000026.1	RiPP-like	-	
	BMHE01000044.1	RiPP-like	-	
	BMHE01000059.1	proteusin	-	
	BMHE01000086.1	NRPS	bacillibactin	53
P. aceris	JAGGKV010000001.1	LAP/RiPP-like	-	
	JAGGKV010000001.2	terpene	-	
	JAGGKV010000001.3	RiPP-like	-	
	JAGGKV010000006	proteusin	-	
	JAGGKV010000008	terpene	-	
	JAGGKV010000026	siderophore	-	
	JAGGKV010000028	lassopeptide	paeninodin	100

Table S5. Peptide of strain SS4^T using Norine database

Total	Similarity	Peptide	Activity
2	0.303	kurstakin C12	antimicrobial
2	0.303	kurstakin C12:0-OH(3)	antimicrobial
2	0.303	kurstakin C14:0-OH(3)	antimicrobial
2	0.303	kurstakin iC11	antimicrobial
2	0.303	kurstakin iC12	antimicrobial
2	0.303	kurstakin iC13	antimicrobial
2	0.303	kurstakin iC13:0-OH(3)	antimicrobial
1	0.571	kahalalide G	unknown
1	0.537	microginin FR3	protease inhibitor
1	0.537	microginin FR5	protease inhibitor
1	0.534	apramide A	unknown
1	0.499	gratisin	antimicrobial
1	0.462	fengycin A iC17	antimicrobial, surfactant
1	0.433	pseudobactin A	siderophore
1	0.458	pyoverdin C	siderophore
1	0.458	pyoverdin BTP16	siderophore
1	0.496	isopyoverdin 90-33	siderophore
1	0.394	callipeltin B	toxin
1	0.394	cupolamide A	toxin
1	0.360	syringopeptin SC 1	antimicrobial, toxin
1	0.360	syringopeptin SC 2	antimicrobial, toxin
